# Addressing food insecurity: An exploration of wheat production expansion

**DOI:** 10.1371/journal.pone.0290684

**Published:** 2023-12-13

**Authors:** Amir Dadrasi, Mehrdad Chaichi, Alireza Nehbandani, Abdollatif Sheikhi, Fatemeh Salmani, Ahmad Nemati

**Affiliations:** 1 Department of Agronomy, Agriculture College, Vali-e-Asr University of Rafsanjan, Rafsanjan, Iran; 2 Department of Seed and Plant Improvement Research, Hamadan Agriculture and Natural Resources, Research and Education Center, Agriculture Research, Education and Extension Organization, Hamadan, Iran; 3 Department of Plant Production, Gorgan University of Agricultural Sciences, Gorgan, Iran; 4 Department of Horticulture, Agriculture College, Vali-e-Asr University of Rafsanjan, Rafsanjan, Iran; 5 Hamadan Agriculture and Natural Resources, Research and Education Center, Agriculture Research, Education and Extension Organization, Hamadan, Iran; ICAR Indian Institute of Wheat and Barley Research, INDIA

## Abstract

Wheat plays a crucial role in global food security, serving as a vital food crop that feeds billions of people worldwide. Currently, Russia and Ukraine are responsible for exporting approximately 25% of the world’s wheat, making any issues in these regions a cause for concern regarding global wheat supply. The problems faced in these areas have led to a surge in wheat prices worldwide. Consequently, it becomes necessary to explore alternative regions that can compensate for the decline in wheat production and supply. This study focuses on wheat production and yield in major producing countries, utilizing the GYGA (Global Yield Gap Atlas) protocol for predictions. The findings reveal a global wheat production gap of 270,378,793 tons. Notably, the largest gap in irrigated wheat production exists in countries like China, India, Pakistan, Turkey, Iran, Afghanistan, Uzbekistan, Egypt, and Azerbaijan. Additionally, the rainfed wheat production gap on a global scale amounts to 545,215,692 tons, with Russia, the USA, Kazakhstan, Australia, Ukraine, China, Turkey, Canada, India, and France having the most significant production gaps. Through boundary line analysis, specific criteria were identified for suitable areas of irrigated and rainfed wheat cultivation. For irrigated conditions, the temperature range of 3000 to 7000 GDD (Growing Degree Days) and a temperature seasonality of 3 were determined as favorable. Under rainfed conditions, the suitable areas encompass a temperature range of 2000 to 4000 GDD, an aridity index exceeding 600, and a temperature seasonality of 2. Thirteen countries possess extensive agricultural land within the climatic codes favorable for irrigated wheat cultivation. Approximately 50% of the agricultural lands within these countries, corresponding to the total arable area for irrigated wheat, fall within the climatic codes 3403, 5403, 5303, 4303, 5503, 5203, 3503, 3303, and 4103. China, the United States, Ukraine, Russia, and Iran are the top five countries with favorable lands for irrigated wheat cultivation. Similarly, fourteen countries have significant agricultural lands within the favorable climatic codes for rainfed wheat cultivation. Around 52% of the agricultural lands within these countries are within the climatic codes 3702, 2702, 2802, and 4602. France, Germany, Britain, Poland, and Denmark possess the highest potential to expand rainfed wheat cultivation areas within these favorable climate codes, with respective areas of 2.7, 2.6, 1.6, and 0.9 million hectares. According to the study, the North China Plain emerges as a primary region for increasing irrigated wheat production, both in terms of cultivated area and yield potential. For rainfed conditions, the European continent stands out as a significant region to enhance wheat production.

## Introduction

As a significant and ancient crop in the global food supply, wheat serves as a primary source of energy in the human diet worldwide [1]. As reported by the FAO, the projected demand for wheat by 2050 is approximately 840 million tons, while the present production is around 750 million tons [2]. In order to fulfill this demand, developing countries will have to increase wheat production by 77%, and a majority of this growth will need to be accomplished through improved yield per unit area [2].

Currently, the unpleasant events in Russia and Ukraine, as the two major producers of wheat in the world, have led to a new level of concern. In 2021, Russia produced approximately 85.9 million metric tons of wheat, representing around 13.7% of global wheat production [2]. This substantial production volume solidifies Russia’s position as one of the leading wheat producers worldwide. Similarly, Ukraine produced approximately 30.5 million metric tons of wheat in 2021, accounting for approximately 4.9% of global wheat production. Ukraine’s contribution to global wheat production highlights its significance in the global market. Together, Russia and Ukraine accounted for approximately 18.6% of global wheat production in 2021, making them significant contributors to the global wheat supply. the policy of the FAO is not to produce wheat excessively. The FAO’s policies and initiatives related to wheat production are focused on promoting sustainable agriculture and food security, which includes efforts to increase wheat production in a sustainable manner. This means that the FAO aims to increase wheat production to meet the growing demand for food, while also ensuring that natural resources are conserved and that the production methods used are environmentally sustainable. The FAO recognizes the importance of balancing the need for increased food production with the need to protect the environment and promote sustainable development [3]. Furthermore, there is an immediate necessity to improve productivity through agronomic (water, nutrients, weed management, etc.), genetic and physiological interventions along with resource conservation technologies. Research into climate change monitoring, adaptation, and crop modeling for yield predictions will be beneficial in meeting future requirements [4].

According to the FAO survey report, wheat imports in North Africa are projected to rise from 22.3 million tons in 2010 to 51.4 million tons in 2050, despite a decline in per capita wheat consumption [5]. In the Middle East, imports from small wheat producers are expected to double from 14.4 million tons to 29.5 million tons. In sub-Saharan Africa, where the population and per capita wheat consumption are growing the fastest, wheat imports are expected to increase by 23.1 million tons by 2050. Indonesia’s wheat imports are projected to increase by 30% to 7.1 million tons in 2050 [5], while the Philippines’ wheat imports will rise to 4.5 million tons by 2050 due to a growth in domestic demand [6]. Brazil will need to increase its wheat imports from 6.5 million tons in 2010 to 10.5 million tons in 2050 to meet the domestic demand. India needs to produce around 140 million tons of wheat by 2050 to keep up with population growth. Mexico’s wheat production is expected to remain constant until 2050 due to negative population growth rate [7].

According to [5], the primary wheat exporting countries in the world are the United States of America, Canada, Russia, Ukraine, Australia, the Black Sea region, Europe, and Argentina. However, these countries are predicted to have a minimal or even negative population growth until 2050. In contrast, tropical and subtropical countries where wheat production is limited are expected to have the highest population growth. Weigand [5] also states that even if significant wheat imports from China are not forecasted, global wheat trade is projected to increase to 240 million tons or more by 2050.

Currently, wheat accounts for a significant portion of the global grain trade, making up one-third of the total trade [8]. The continuous growth of wheat exports has led to notable implications for all segments of the industry, especially in countries where such exports have intensified. Nevertheless, there exist major challenges to maintaining wheat production in the future due to the ever-increasing population, diminishing purchasing power, and reduction in cultivable land and water resources. Additionally, changes in climate conditions, like increased temperatures and fluctuations in the amount and distribution of rainfall, as well as unforeseeable events such as war, make it increasingly difficult to meet the growing demand for wheat [9]. According to [10], to meet the projected demand for food, an annual growth of 2 to 3 percent in the food supply is necessary. However, the yield of major grains such as wheat, corn, and rice has grown at a rate of less than one percent per year in the last decade [10]. This slow growth poses a significant challenge because by 2050, many countries with inadequate food reserves will experience further population growth, which will increase the pressure to increase their food supplies [11]. Fischer and Connor [12] predicted that based on population projections and wheat production and consumption growth rates, the regions of North Africa, the Middle East, Sub-Saharan Africa, Indonesia, the Philippines, Brazil, and Mexico will experience an increase of more than double in wheat imports.

To address the current wheat production crisis, increasing production is crucial, which can be accomplished by either expanding the area under wheat cultivation or by enhancing the yield per unit area. Sustainable resilience strategies include expanding wheat production to new areas, such as Sudan, Kenya, Nigeria, and Tanzania, which have a comparative advantage for wheat cultivation [13]. However, it is important to ensure that the food security of other crops is not jeopardized by developing wheat lands in suitable areas, while prioritizing wheat cultivation in regions with less yield gap and higher production [14]. Therefore, expanding wheat cultivation to areas with high yield potential and increasing its concentration in such areas is essential to supply the growing population’s demand for wheat in the current and future conditions.

There are numerous organizations that have created maps showcasing the cultivated areas for major crops, including wheat, on a global scale. One such resource is the MapSpam (https://dataverse.harvard.edu/dataset.xhtml?persistentId=doi:10.7910/DVN/PRFF8V) website, which provides maps of cultivated areas in various formats that are compatible with ArcGIS software [15]. These maps, along with the data provided by the GYGA (https://www.yieldgap.org) website, can be utilized to identify regions that are suitable for cultivation. To identify areas with the potential for high wheat production and yield, as well as suitable lands for wheat cultivation, this study utilized information from various sources. These sources included 1- reported results of irrigated and rainfed wheat cultivation in different countries using the GYGA protocol and 2- maps from the MapSpam website, which served as input files for ArcGIS software for both irrigated and rainfed wheat.

## Materials and methods

To assess the potential for increasing wheat production by estimating the yield gap, production gap, and extensible harvested area, several pieces of information are required. These include actual and potential yield and production figures, as well as the current wheat harvested area and the total land area that could potentially be used for wheat cultivation. Each of these sections will be described in detail in the following section.

### Harvested area, actual yield, and production

The global maps of wheat cultivation area were acquired from the MapSpam site, to determine the yield gap and potential yield of crops in several countries [16–19]. However, to identify the areas suitable for wheat cultivation, a complete map is not available. To address this issue, the total cultivated area of other crops with similar growing seasons, including barley, canola, lentils, and peas, were also considered. By subtracting the area under wheat cultivation in the current scenario from the total area under cultivation of similar crops, the areas with potential for wheat cultivation were identified separately for irrigated and rainfed conditions. Information about actual yield and production in irrigated and rainfed conditions was also obtained from the MapSpam site. All these maps were available in the shapefile format on the MapSpam website, which was prepared specifically for this research using the ArcGIS software.

### Estimating potential yield and production of wheat in the world as well as the yield and production gap of wheat worldwide

**Fig 1:**
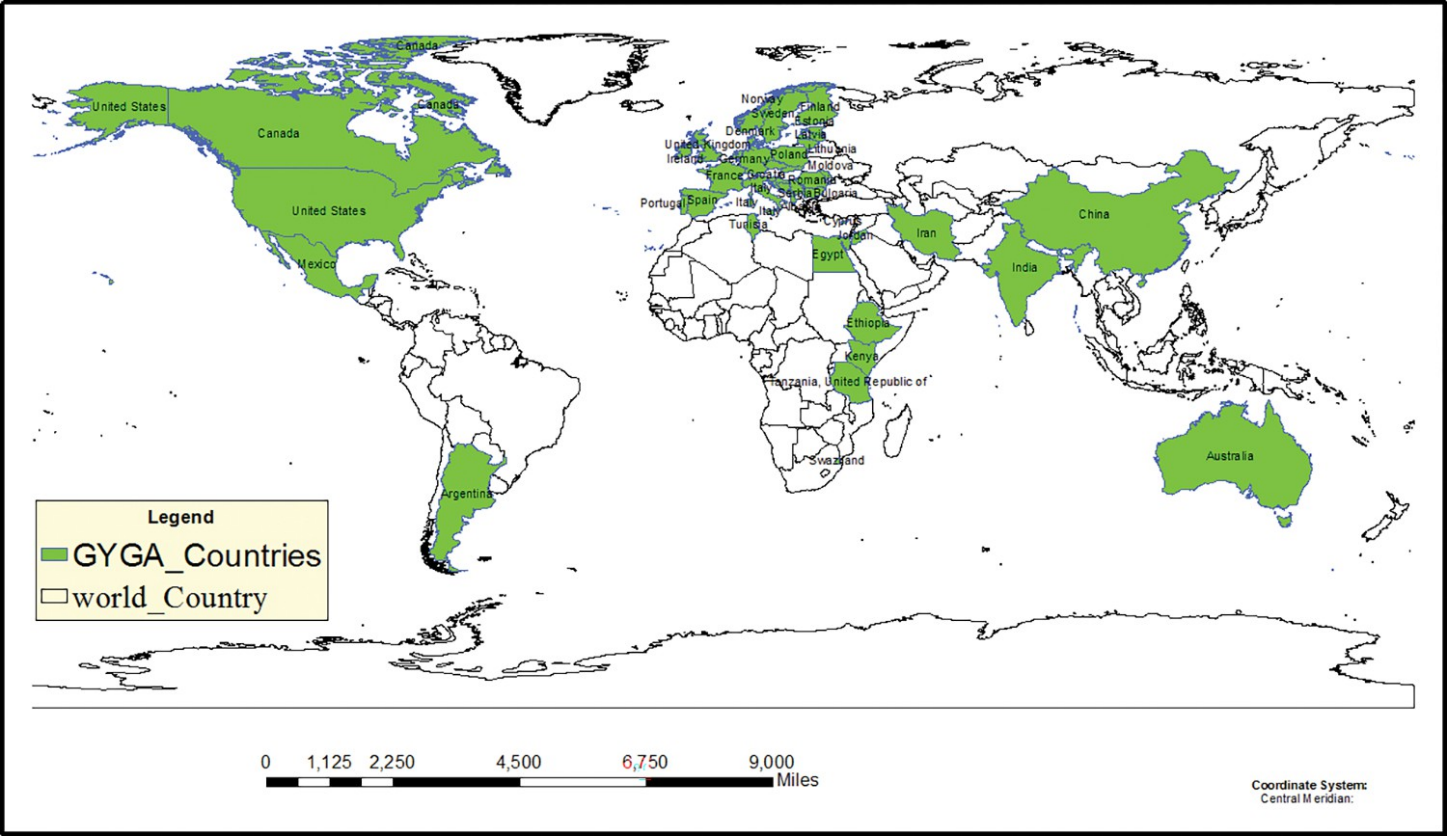
The map highlights the countries which have utilized the Global Yield Gap Atlas (https://www.yieldgap.org) to calculate the potential yield, actual yield, and yield gap for wheat, and they are marked in green. A total of 49 countries have extracted this information according to the GYGA protocol. Major wheat production areas were obtained from GYGA website.

To estimate the potential yield and production, as well as the yield and production gap of wheat worldwide, the Global Yield Gap Atlas (GYGA) was relied upon due to the absence of such data on the MapSpam website and other databases. The GYGA protocol was designed to estimate the potential and yield gap of strategic crops at the national level, taking into account various agro-climatic zones (CZs), Reference Weather Stations (RWS), buffer zones, and soil types in each buffer zone. More information is available in (https://www.yieldgap.org). Information for wheat in both irrigated and rainfed conditions was estimated for around 49 countries, and the outcomes were obtained from the Global Yield Gap Atlas website. Similar climate conditions were used to determine the potential yield, irrespective of the political boundaries of countries. In Fig 1, the countries that estimated wheat yield potential using the GYGA protocol are illustrated. The information was utilized in this research to estimate the potential yield of irrigated and rainfed wheat worldwide, and production potential values were calculated using the cultivation area map and the yield potential of irrigated and rainfed wheat globally. Production potential values were calculated by combining the cultivation area map with the global yield potential of irrigated and rainfed wheat. To estimate the yield and production gaps, two crucial factors were required: actual values and potential values. The GYGA website provided information on potential yield and production separately for irrigated and rainfed wheat, considering the critical climatic conditions necessary for the crop’s cultivation. This data was utilized to generalize the findings to regions worldwide that share similar climatic conditions.

### Identification suitable climate zone for wheat production in the world

The actual and potential yields for both irrigated and rainfed cultivation systems were obtained from the GYGA website for each climate zone in every country. The next step involved setting a regression equation between the actual and potential yields and the climate zone code (which comprises three parameters) through boundary linear analysis. Subsequently, the equations were used to determine the appropriate climate conditions. The GYGA climate maps, codes, and naming conventions are fully explained as follows:

#### GYGA climate map

The map classifies climatic zones using data from three variables, namely growing degree days (GDD) with a base temperature of zero degrees Celsius, temperature seasonality, and annual aridity index (AI). Eq 1 is used to calculate the GDD.


$$GDD=\sum_{i}^{n}t_{i}$$ (1)

Where, GDD is the unit of temperature throughout the year in terms of day degrees; n, the number of days during the year, which is 365 days in normal years and 366 days in leap years; i, day of the year; t_i_, the average daily temperature on a day i. If the average daytime temperature of the year is below zero degrees Celsius, t_i_ is considered zero for that day. Obviously, for a climatic zone, the higher GDD value indicates that the average temperature of that climatic zone is higher throughout the year.

The temperature seasonality is the standard deviation of average monthly temperatures that is obtained from Eq 2:

$$Temperature\;Seasonality=\sqrt{\sum_{m}^{12}\frac{{\left(t_{m}-t_{avr}\right)}^{2}}{12}}$$ (2)

Where, temperature seasonality is the coefficient of temperature fluctuations throughout the year; t_m_: average temperature in the month m in degrees Celsius; t_avr_: the average temperature throughout the year in degrees Celsius. The larger seasonal temperature fluctuations coefficient for a climatic zone shows that temperature fluctuations are higher throughout the year in that climatic zone. In simpler terms, the difference between the coldest and warmest months of the year is greater.

The third index used for zoning by the extrapolation method of the Global Atlas of yield gap is an aridity index derived from Eq 3.


$$AI=\frac{MAP}{MAE}$$ (3)

Where AI is annual aridity index; MAP: Average annual rainfall in millimeters; MAE: Average annual evaporation in millimeters. According to this equation, in a climatic zone, the smaller the amount of AI, the drier the zone (www.yieldgap.org/web/guest/cz-ted). The information source for these variables is the website www.yieldgap.org/web/guest/cz-ted.

#### Boundary line analysis

In order to determine the optimal global climatic zones for growing wheat, data on actual and potential yields for both irrigated and rainfed conditions were utilized. Specifically, the relationship between actual and potential yields and three indices (Growing Degree Days, temperature seasonality, and annual aridity index) were analyzed using boundary line analysis to identify the desired range for each indicator. It should be noted that while there is no universally accepted protocol for boundary line analysis, in some instances, researchers may fit the boundary line to the data at their discretion [20]. The steps involved are as follows, drawing upon the work of Makowski et al. [20], Nehbandani et al. [21], and Yousefian et al. [22], as well as Smith and Hardie [23]:

1. Review the data dispersion diagram: A dispersion diagram, also known as an XY diagram, was constructed, with crop yield as the dependent variable and one of the management variables (GDD, temperature seasonality, or AI) as the independent variable. This step allowed for visualizing the data cloud and aided in selecting an appropriate function to represent the upper edge of the data cloud.
2. Select data points from the upper edge of the data cloud for curve fitting: Data points were chosen from the upper edge of the data cloud by visual inspection or using advanced statistical methods. In this study, we selected data points from the upper edge of the data cloud through visual inspection and plotted a suitable function based on these points.
3. Select an appropriate function: The final step involved choosing an appropriate function based on the selected data points from the previous step. This process resulted in a model that expresses the maximum efficiency response to different levels of the independent variable being analyzed.

## Results

### Determination area with high production and yield gap

#### Irrigated wheat

**Fig 2:**
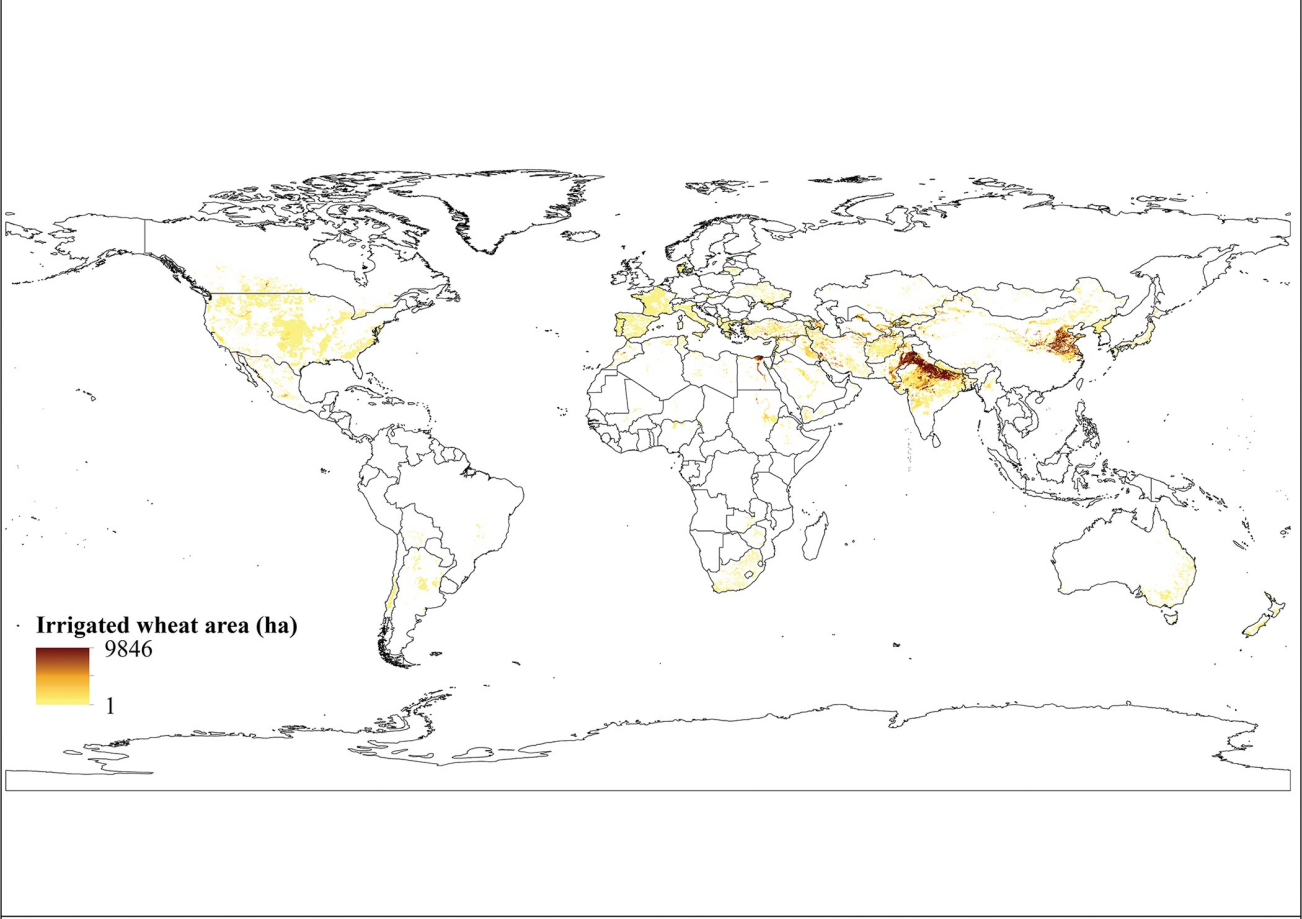
Worldwide cultivation area of irrigated wheat. In this map areas with higher cultivation levels are shown in dark brown and areas with less cultivation levels are exhibited in yellow color. Irrigated wheat cultivation areas were obtained from SPAM 2010.

*Harvested area*. According to the findings, the global extent of wheat cultivation under irrigation amounts to approximately 60 million hectares (59,890,480 hectares), which accounts for approximately 27.2% of the total global wheat cultivation area. Notably, the countries with the most significant levels of cultivation are India, China, Pakistan, Iran, Turkey, Afghanistan, Egypt, and the United States, as shown in Table 1. Additionally, Fig 2 presents a graphical representation of the worldwide area under irrigated wheat cultivation.

**Table 1:** Harvested area, actual yield, actual production, potential yield, potential production, yield gap, and production gap in the main countries producing irrigated wheat. The data used in this study was obtained from the GYGA and MapSpam websites in 2022. The GYGA dataset covers the last 15 years for rainfed condition and 10 years for irrigated condition.

Country	Harvested area (ha)	Actual yield (t/ha)	Potential yield (t/ha)	Yield gap (t/ha)	Actual production (ton)	Potential production (ton)	Production gap (ton)
China	15009053	5.50	11.93	6.43	82556099	179101683	96545584
India	22232015	3.28	6.52	3.24	72882744	144862554	71979810
Pakistan	6199646	3.58	7.51	3.93	22186026	46550403	24364377
Egypt	1295385	6.18	9.38	3.20	8010773	12156348	4145575
Iran	2547125	4.67	8.59	5.52	7831436	21882465	14051029
United States	1219723	6.00	9.34	3.33	7319320	11386814	4067494
Turkey	1629788	3.61	9.55	5.94	5879621	15566514	9686893
Uzbekistan	1142313	5.10	9.21	4.12	5822195	10524021	4701826
Mexico	608675	5.90	9.04	3.14	3588423	5501758	1913335
Afghanistan	1397204	2.47	8.64	6.17	3452550	12072625	8620075
Syria	567734	4.12	7.45	3.34	2336319	4231875	1895556
Canada	366639	5.28	7.15	1.87	1936112	2622618	686506
Morocco	246255	6.95	7.31	0.35	1712682	1800077	87395
Iraq	638170	2.50	7.18	4.67	1598130	4580028	2981898
Azerbaijan	622926	2.54	9.12	6.58	1582587	5679199	4096612
Nepal	704466	2.17	7.20	5.03	1528920	5073371	3544451
Turkmenistan	642887	2.17	8.47	6.29	1397560	5442452	4044892
Saudi Arabia	167570	6.63	8.35	1.72	1110786	1398956	288170
Bangladesh	300568	2.62	6.68	4.06	786302	2007873	1221571
Kyrgyzstan	309434	2.53	8.39	5.86	783304	2597020	1813716
**World (Sum/Aver)**	59890480	4.72	8.94	4.22	242389426	512768219	270378793

**Fig 3:**
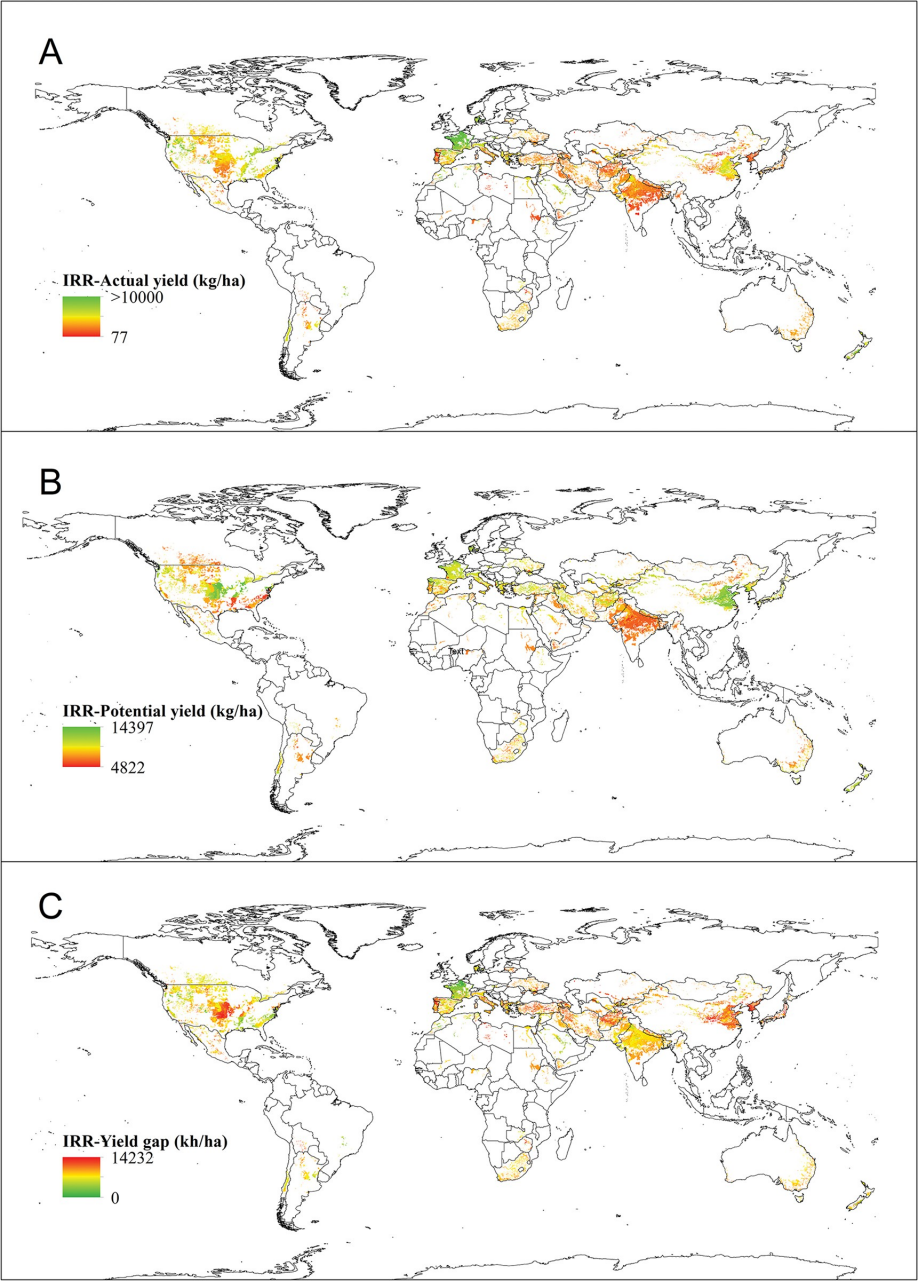
Worldwide actual yield (A), potential yield (B) and yield gap (C) of irrigated wheat. In 3A and 3B, the highest and lowest actual and potential yield values are shown in green and red color, respectively. In 3C, on the contrary, the highest values of production gap are shown in red color and the lowest values are exhibited in green color. The actual yield of irrigated wheat (A) was obtained from SPAM 2010 and the potential yield (B) of irrigated wheat was retrieved from GYGA website.

*Actual yield*. According to Table 1 and Fig 3A, the global average yield of irrigated wheat is 4.72 tons per hectare. Morocco, Saudi Arabia, Egypt, and the United States boast the highest yields at 6.95, 6.63, 6.18, and 6 tons per hectare, respectively. On the other hand, China, India, and Pakistan have lower actual yields of 5.50, 3.28, and 3.58 tons per hectare, respectively.

*Potential yield*. Table 1 and Fig 3B display the potential yield data for various countries. The global average for potential yield is 8.94 tons per hectare. Notably, China, Turkey, Egypt, the USA, Uzbekistan, Azerbaijan, and Mexico have the highest potential yields, with values of 11.93, 9.55, 9.38, 9.34, 9.21, 9.12, and 9.04 tons per hectare, respectively (Table 1, Fig 3B).

*Yield gap*. The disparity between actual and potential yield of irrigated wheat is illustrated in Fig 3C. Table 1 displays the global yield gap, which is currently measured at 4.22 tons per hectare. Effective farm management has the potential to decrease this yield gap. In areas with low production despite high yield potential, altering cultivation patterns and relocating to more suitable areas can balance the production gap. For instance, countries such as Azerbaijan, China, Turkmenistan, Afghanistan, Turkey, Kyrgyzstan, and Iran have a yield gap exceeding the global average. Relocating crop production from water-scarce areas to water-rich regions can offset a portion of the production gap (Table 1).

**Fig 4:**
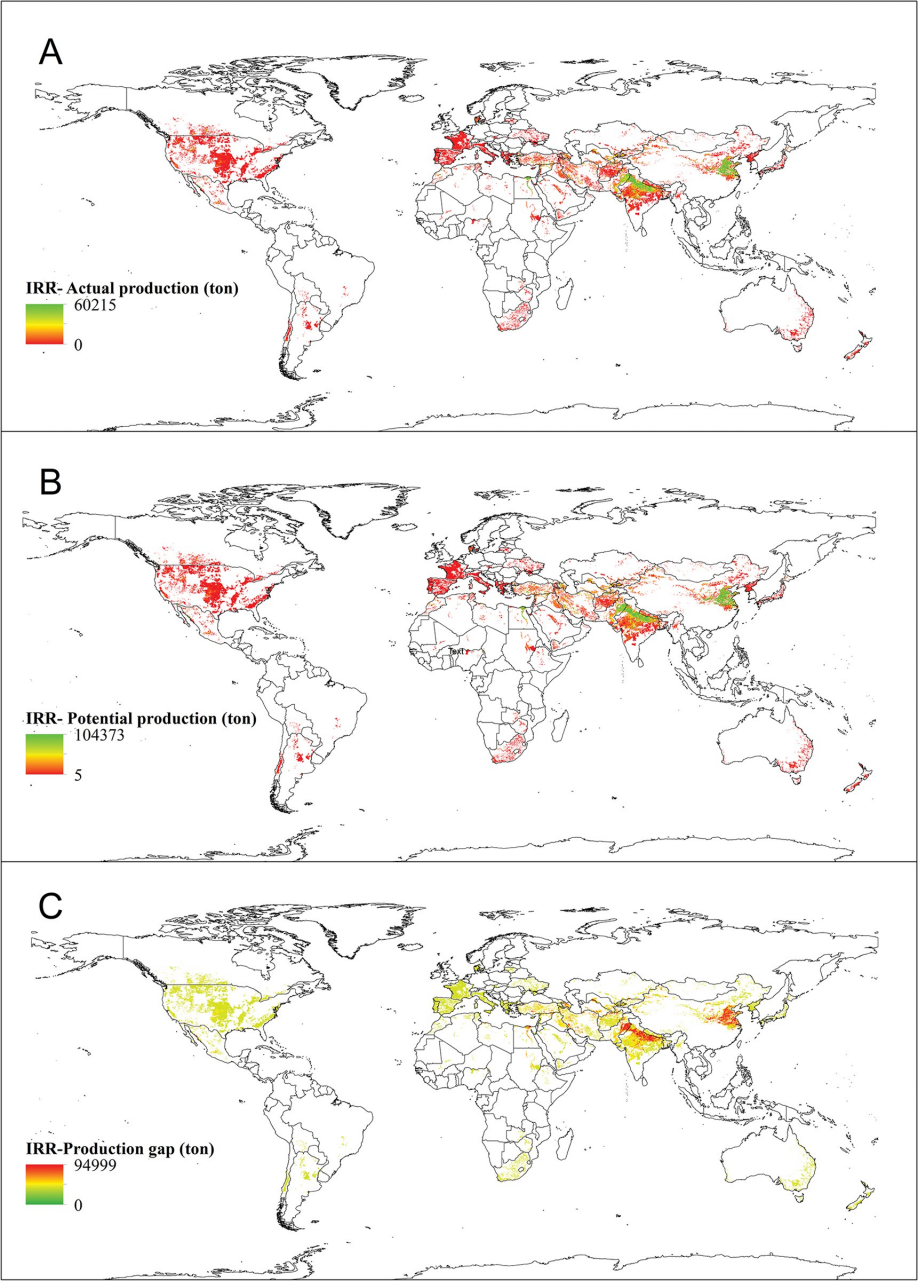
Worldwide actual production (A), potential production (B) and production gap (C) of irrigated wheat. In 4A and 4B, the highest and lowest actual and potential yield values are shown in green and red color, respectively. In 4C, on the contrary, the highest values of production gap are shown in red color and the lowest values are exhibited in green color. The actual production of irrigated wheat (A) was obtained from SPAM 2010 and the potential production (B) of irrigated wheat was retrieved from GYGA website.

*Actual production*. The global cultivated area and corresponding actual yield, and the product of these two factors is known as the global actual production (Table 1). Specifically, for irrigated wheat, the global actual production is approximately 242,389,426 tons (≈ 36% of the total global wheat actual production). This can be visualized in Fig 4A, which depicts the actual production of irrigated wheat on a global scale.

*Potential production*. Global potential production, which is the result of the cultivated area and potential yield, amounts to 512,768,219 tons for irrigated wheat (≈ 34% of the total global wheat potential production), as indicated in Table 1. The potential production of irrigated wheat across the globe is illustrated in Fig 4B, revealing that countries with high potential yields also have high potential production.

*Production gap*. According to the findings, irrigated wheat has a global production gap of 270,378,793 tons (Table 1), that represents around 33% of the total production gap. The corresponding data is presented in Fig 4C, which illustrates the production gap at a global scale. Notably, China, India, Pakistan, Turkey, Iran, Afghanistan, Uzbekistan, Egypt, and Azerbaijan are among the major wheat-producing nations with the highest production gaps.

#### Rainfed wheat

**Fig 5:**
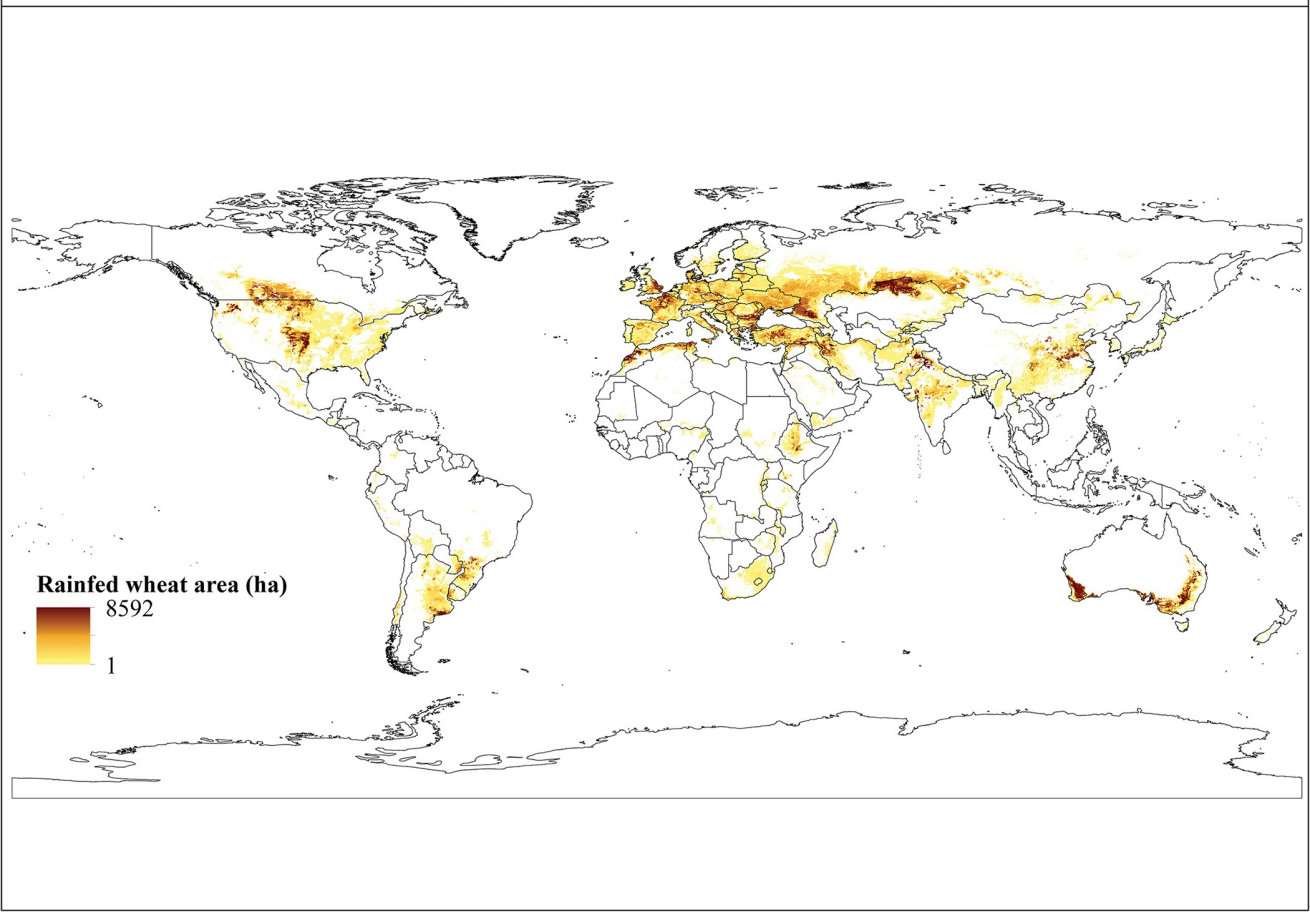
This map displays the global cultivation area of rainfed wheat, with darker shades of brown indicating regions of higher cultivation and lighter shades of yellow indicating lower levels of cultivation. Cultivation area of rainfed wheat was retrieved from SPAM 2010.

*Harvested area*. The majority of wheat worldwide is produced under rainfed conditions, with a total global cultivated area of 160,401,993 hectares (Table 2), which accounts for approximately 72.8% of the total global wheat cultivation area. The countries with the highest levels of rainfed wheat cultivation include Russia, the United States, Kazakhstan, Australia, China, Canada, Ukraine, Turkey, India, and France, as indicated in Table 2. The area under cultivation of rainfed wheat in all countries of the world is illustrated in Fig 5.

**Table 2:** Summarizes information on rainfed wheat production in major producing countries, including the size of cultivated land, actual and potential yields, actual and potential production, as well as the yield and production gaps. The data used in this study was obtained from the GYGA and SPAM websites in 2022. The GYGA dataset covers the last 15 years for rainfed condition and 10 years for irrigated condition.

Country	Harvested area (ha)	Actual yield (t/ha)	Potential yield (t/ha)	Yield gap (t/ha)	Actual production (ton)	Potential production (ton)	Production gap (ton)
Russia	24324779	2.18	6.59	4.41	53019542	160274397	107254855
United States	18051715	2.82	5.59	2.77	50866808	100854212	49987404
France	5553901	6.60	9.62	3.02	36666728	53441511	16774783
China	9217388	3.60	6.68	3.08	33184047	61568951	28384904
Germany	3254936	7.37	9.64	2.27	24000727	31384962	7384235
Australia	13485339	1.73	4.30	2.57	23278253	58001895	34723642
Canada	8452072	2.74	5.28	2.53	23196109	44591295	21395186
Ukraine	6499075	3.05	8.03	4.98	19830052	52189390	32359338
Kazakhstan	13491272	1.18	4.23	3.06	15866638	57094982	41228344
Turkey	6417578	2.30	6.19	3.90	14742847	39741867	24999020
United Kingdom	1882954	7.79	10.24	2.45	14661706	19278409	4616703
Argentina	4005577	2.73	4.98	2.24	10951227	19937256	8986029
India	6206059	1.55	4.87	3.31	9644556	30206809	20562253
Poland	2327511	4.09	9.30	5.21	9515067	21639208	12124141
Italy	1754358	3.69	6.99	3.29	6480476	12257519	5777043
Romania	2083099	2.91	7.71	4.79	6069931	16056331	9986400
Brazil	2243092	2.51	5.84	3.33	5620283	13092283	7472000
Spain	1842303	2.99	6.23	3.24	5513067	11476168	5963101
Denmark	664715	6.83	9.14	2.31	4542363	6076084	1533721
Czech Republic	840940	5.31	9.27	3.96	4464827	7791771	3326944
**World (sum/ave)**	160401993	2.68	6.58	3.90	431375851	976591543	545215692

**Fig 6:**
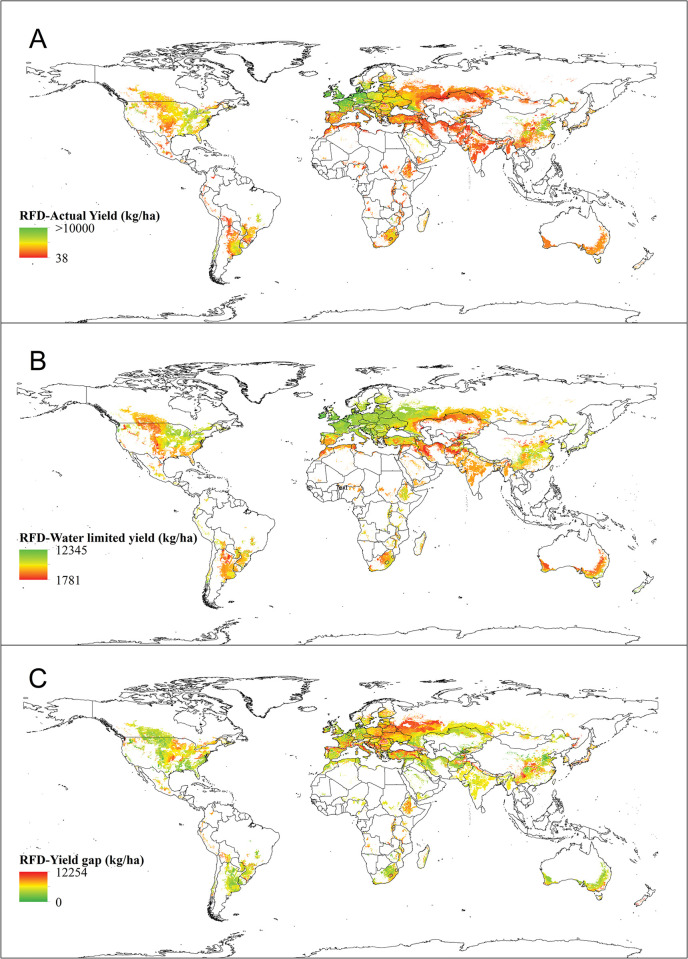
The figures display the actual yield (A), potential yield (B), and yield gap (C) of rainfed wheat worldwide. The highest and lowest values for actual and potential yield are represented in green and red, respectively, in 6A and 6B. Conversely, in 6C, the highest production gap values are highlighted in red, while the lowest values are indicated in green. The actual yield of rainfed wheat (A) was retrieved from SPAM 2010 and the potential yield (B) of rainfed wheat was obtained from GYGA website.

*Actual yield*. According to Fig 6A and Table 2, the countries with the highest actual yields of rainfed wheat are the United Kingdom, Germany, Denmark, France, the Czech Republic, Poland, Italy, China, and Ukraine. The estimated world average yield for rainfed wheat is 2.68 tons per hectare.

*Potential yield*. The study revealed that rainfed wheat had an estimated global potential yield of 6.58 tons per hectare. The countries with the highest potential yields for rainfed wheat were the United Kingdom, Germany, France, Poland, the Czech Republic, Denmark, Ukraine, Romania, Italy, China, and Russia. These countries had potential yields ranging from 1,781 to 12,345 kg/ha, as shown in Fig 6B and Table 2.

*Yield gap*. The rainfed wheat’s worldwide average yield gap was 3.90 tons per hectare, and the countries with the most significant yield gaps were Poland, Ukraine, Romania, Russia, Czech Republic, Turkey, Brazil, and India (Table 2 and Fig 6C).

*Actual production*. Russia, USA, France, China, Germany, Australia, Kazakhstan, Canada, Ukraine, Turkey, England, and Argentina are among the countries with the highest actual production of rainfed wheat, contributing to the total global production of 431,375,851 tons (Table 2), that represents ≈ 64% of the total global wheat actual production.

**Fig 7:**
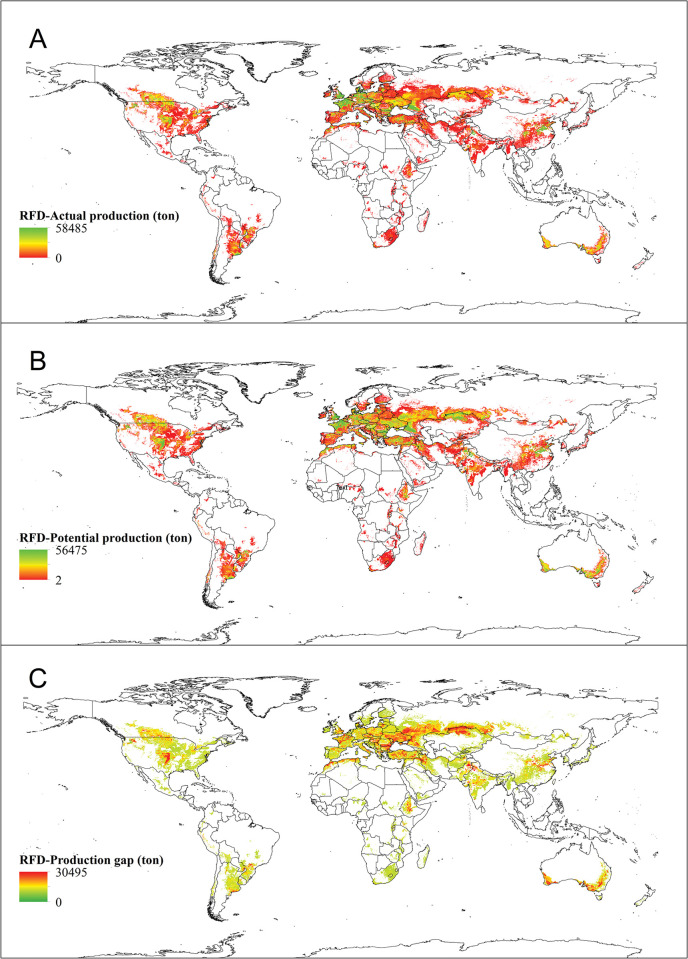
The figures present information on the global production of rainfed wheat, including actual production (A), potential production (B), and the difference between the two (C) referred to as the production gap. 7A and 7B highlight the maximum and minimum actual and potential yields, which are represented in green and red colors respectively. 7C, on the other hand, displays the highest production gap values in red and the lowest in green. The actual production of rainfed wheat (A) was retrieved from SPAM 2010 and the potential production (B) of rainfed wheat was obtained from GYGA website.

*Potential production*. The possible amount of rainfed wheat production worldwide, as well as the area where it is grown, impact its overall yield. As indicated in Table 2, the total global production of rainfed wheat reached 976,591,543 tons, which representing roughly 66% of the total global wheat production potential. Notably, the countries with the highest potential for rainfed wheat production are Russia, the United States, China, Australia, Kazakhstan, France, Ukraine, Canada, Turkey, Germany, India, Poland, Argentina, and the United Kingdom, as depicted in Fig 7B and Table 2.

*Production gap*. By examining the rankings of nations based on their current and future wheat production, one can notice a global discrepancy in output among major wheat-producing countries. This discrepancy can be attributed to the difference in yield (Fig 7C). The global production gap for rainfed wheat amounts to 545,215,692 tons (near to 67% of the total global wheat production gap), with the largest gaps found in Russia, the USA, Kazakhstan, Australia, Ukraine, China, Turkey, Canada, India, and France (Table 2).

### Determining expandable harvest area for wheat

#### Irrigated wheat cultivation

**Fig 8:**
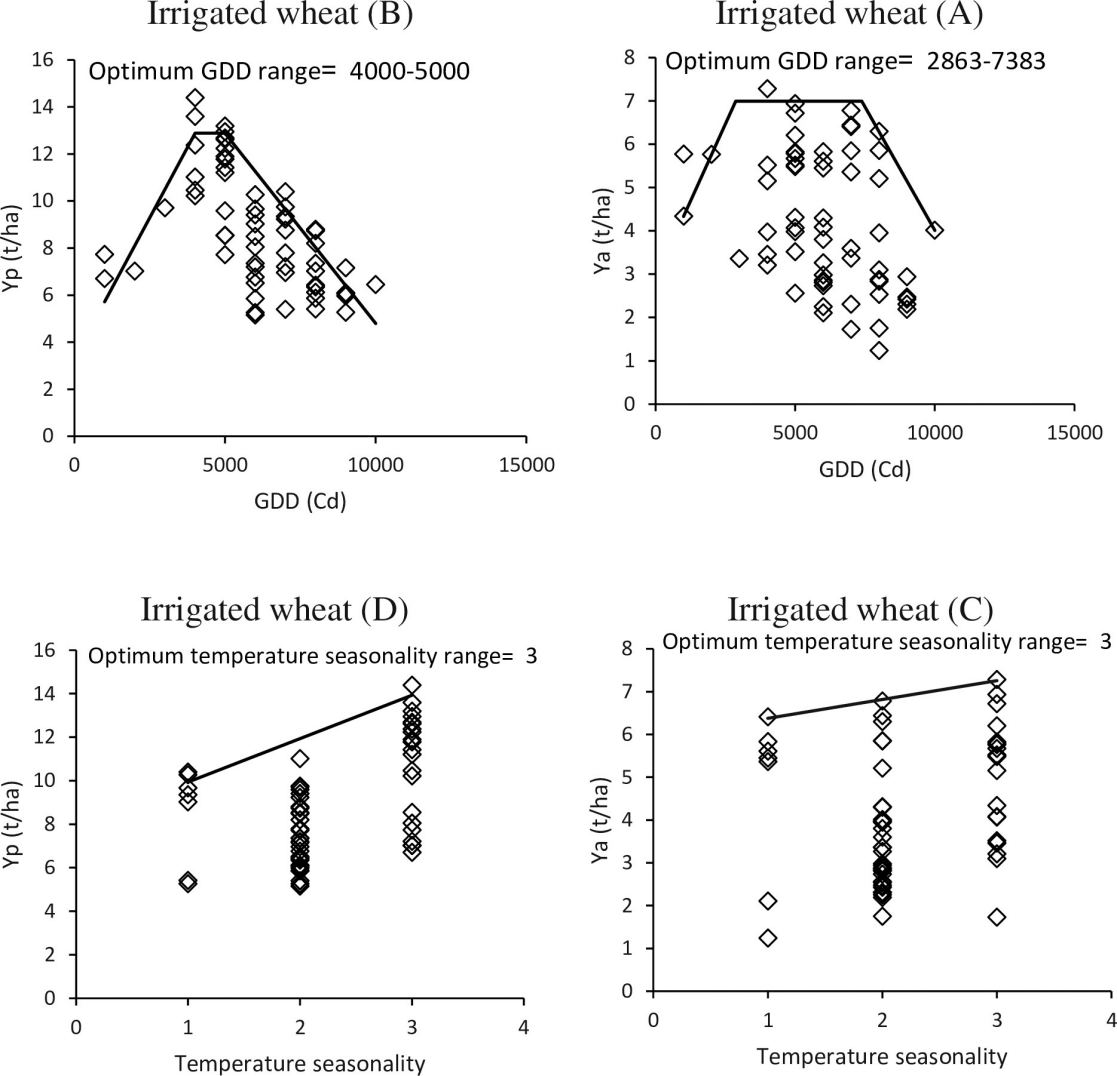
These charts display how the actual yield (Ya) and potential yield (Yp) values vary with the growing degree-day index (A & B) and temperature seasonality index of climate zone code (C & D). A boundary line representing the function fitted maximum yields is shown in black. The data pertains to an irrigated condition.

The relationship between the actual and potential yield of irrigated wheat and the temperature unit was analyzed and the data revealed that the boundary line follows a dent-like function, as shown in Fig 8A and 8B. To achieve maximum potential yield, a temperature range of 4000 to 5000 growth degree days (GDDs) is required (Fig 8A), while achieving maximum actual yield requires a range of 2876 to 7383 GDDs (Fig 8B). Thus, the optimal range of temperature units for achieving maximum yield in irrigated conditions is considered to be between 3000 to 7000 GDDs.

The study found no significant relationship between actual and potential yield and aridity index, which is the mean annual rainfall divided by the average annual transpiration evaporation. This can be justified as rainfall in irrigated conditions has a minimal impact on yield management due to unrestricted water conditions. However, there is a logical relationship between temperature seasonality, which indicates the standard deviation of the average of 12-month the year, and actual and potential yield. The highest values for both actual and potential yield were observed with code 3 (Fig 8C and 8D).

#### Rainfed wheat cultivation

**Fig 9:**
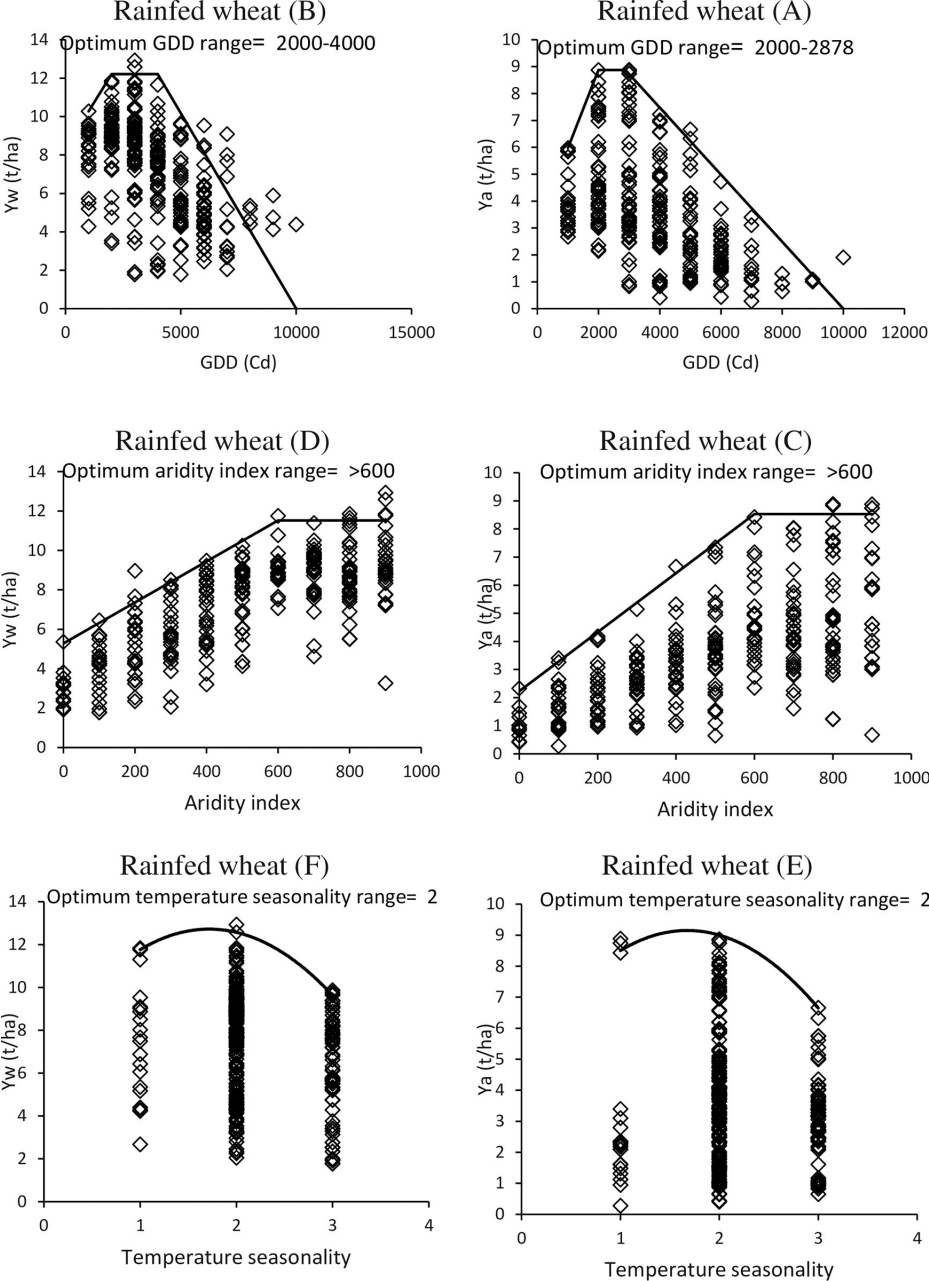
The comparison of real crop yield (Ya) and the maximum yield potential under limited water supply (Yw) in relation to the growing degree-day index (A, B), aridity index (C, D), and temperature seasonality index (E, F) of various climate zones. The black line represents the fitted function boundary line, indicating the maximum possible yield. This comparison is made under rainfed conditions.

According to the analysis of the boundary line between actual yield and water-limited yield potential of rainfed wheat versus temperature unit parameter, the optimal range for rainfed wheat cultivation was found to be between 2000 and 4000 GDDs, as determined by the cloud points below the border lines, which showed the highest values of actual yield and limited water yield compared to other areas (Fig 9A and 9B). The results indicated that the suitable temperature range for rainfed wheat was between 2000 and 4000 GDDs, with 2000 to 2878 GDDs being the most appropriate range for cultivation.

Another critical parameter in the formation of climate codes is the aridity index, which plays a significant role in studying rainfed conditions. The results indicate that climate codes with an aridity index greater than 600 are suitable for cultivating rainfed wheat in terms of both actual yield and limited water. In contrast, codes with an aridity index less than 600 are not ideal for achieving the maximum yield of rainfed wheat due to insufficient rainfall (Fig 9C and 9D).

Studying the sensitivity of temperature seasonality is a crucial aspect in the climate code. The findings of this study revealed that the most optimal conditions for actual and potential yield of rainfed wheat were associated with code 2 when limited water and actual yield were compared to this index. It was observed that more balanced temperature conditions than irrigated wheat were required to achieve maximum yield of rainfed wheat, as shown in Fig 9E and 9F.

The results obtained from the border line analysis indicate that the ideal range of climatic codes for both irrigated and rainfed wheat production are presented in Table 3. For irrigated wheat production, the optimum conditions are identified as the temperature unit range of 3000 to 7000 GDD and a temperature seasonality of code 3. On the other hand, for rainfed wheat production, the most suitable regions are identified with a temperature range of 2000 to 4000 GDD, an aridity index of more than 600, and a temperature seasonality of 2.

**Table 3:** The optimum range of GDD, aridity index, and temperature seasonality for wheat production under irrigated and rainfed conditions.

	Irrigated	Rainfed
**GDD**	3000–7000	2000–4000
**Aridity index**	No limits	>600
**Temperature seasonality**	3	2

### Countries prone to increasing irrigated wheat lands

The study utilized boundary line analysis and yield gap and production gap values to identify countries with high potential for increasing their cultivated areas. Suitable climatic regions in each country were determined to have good potential for expanding their agricultural lands. Thirteen countries were identified with the greatest number of agricultural lands that had favorable climatic codes for producing irrigated wheat (S1A Fig). In these countries, about 50% of agricultural lands (the total area which can be cultivated with irrigated wheat) are in the range of climate codes 3403, 5403, 5303, 4303, 5503, 5203, 3503, 3303, and 4103. Among other countries, China, the United States, Ukraine, Russia, and Iran were ranked first to fifth regarding the existence of favorable lands for irrigated wheat cultivation. Agricultural lands within the range of favorable climatic codes in China, the USA, Ukraine, Russia, and Iran are 24.1, 10.6, 8.0, 7.8, and 5.6 million hectares, respectively. China, Ukraine, Iran, and Russia with 3.7, 3.6, 1.6, and 1.5 million hectares, respectively, have the highest potential to increase the irrigated wheat cultivation area in the favorable climate code. Agricultural lands, wheat cultivated lands, and expandable lands for wheat cultivation in all selected climate codes have been presented separately for each country in S1A–S1C Fig.

### Countries prone to increase rainfed wheat lands

The study found that 14 countries have the most agricultural lands suitable for growing rainfed wheat in favorable climatic conditions (S2A Fig). These countries have approximately 52% of their agricultural lands falling within climate codes 3702, 2702, 2802, and 4602. France, Germany, Britain, Poland, and Denmark were ranked from first to fifth in terms of having the most favorable lands for rainfed wheat cultivation. The areas of agricultural lands with favorable climatic codes in France, Germany, England, Poland, and Denmark are 7.5, 5.2, 3.5, 1.9, and 1.5 million hectares, respectively. France, Germany, Britain, Poland, and Denmark have the highest potential for increasing their rainfed wheat cultivation area in the favorable climate codes, with 2.7, 2.6, 1.6, and 0.9 million hectares, respectively. S2A–S2C Fig shows the agricultural lands, wheat cultivation, and expandable lands for rainfed wheat cultivation separately for each of these countries.

## Discussion

In recent months, the significance of wheat production has heightened due to political issues worldwide, making it a top priority to increase the production of this vital product [24]. One solution is to expand the cultivation area, but another option is to enhance production per unit area [25]. The investigation of wheat’s yield potential in different regions of the world has been conducted in this study. The determination of yield gaps for both irrigated and rainfed wheat crops has been done based on this analysis [26]. Additionally, it is important to pinpoint areas with high production potential to expand the cultivation of this crop. Research has been conducted to investigate the possibility of expanding the cultivation area of this crucial crop by identifying areas with high production potential.

The results, as well as the maps showing the actual yield and cultivation area (Figs 2 and 5), were used to create maps showing the yield gap and potential yield for both irrigated (Fig 2) and rainfed (Fig 6) conditions. These maps also allowed for the creation of production gap maps and potential production maps for both conditions (Figs 4 and 7). It was discovered that China, India, Pakistan, Egypt, Iran, The United States of America, Turkey, Uzbekistan, and other countries mentioned in Table 1 have the expertise to grow irrigated wheat. Upon examining their climatic conditions, it became apparent that these regions have low average precipitation and rely on irrigation for wheat cultivation. As a result, irrigated wheat thrives in these areas, thanks to the availability of water.

The countries listed in Table 2 such as Russia, China, Germany, Australia, Canada, Ukraine, and others, have achieved high yield for rainfed wheat due to adequate rainfall. To determine suitable areas for growing irrigated and rainfed wheat in these countries, boundary line analysis was conducted. This involved assessing climate parameters such as the temperature unit (GDD), aridity index (AI), and seasonal temperature fluctuations (TS) to identify appropriate climate zones in each country. For irrigated wheat cultivation, the temperature unit ranged from 2863 to 7383, with the maximum actual performance observed in this range. However, the potential performance was more restricted, ranging from 4000 to 5000. This indicates that regions with either warmer or colder climates than average can limit the yield potential in irrigated wheat cultivation.

It is clear that insufficient Growing Degree Days (GDD) in cold regions during the growth period, as well as high temperatures and limited wheat C3 crops in hot regions, are both factors that restrict the potential yield of irrigated wheat [27]. However, due to the option of irrigation, this decrease in yield can be largely mitigated compared to rainfed conditions. This suggests that the focus of cultivation should be on climates with temperature units ranging from 4000 to 5000 in order to achieve maximum yield, with the yield gradually decreasing as the temperature unit moves away from this range. Meanwhile, the highest potential and actual yield for rainy conditions occurs in the range of temperature unit between 2000 and 4000. Therefore, it can be mentioned that the moisture availability factor is preferable to the temperature unit factor under rainfed conditions, and the limiting factor for production under these conditions is the humidity, not the temperature unit [28]. The evaluation of results also shows that the aridity index has no significant relationship with the potential and actual yield of irrigated wheat, as this index is not a limiting factor in irrigated conditions. For rainfed conditions, an aridity index above 600 is the most favorable condition for achieving maximum potential and actual yield, as this means that there is more rainfall compared to annual evapotranspiration, providing optimal growth conditions for wheat [29]. This means that under rainfed conditions, environmental factors such as GDD and seasonal temperature fluctuations are restricted by the aridity index.

Fig 8 demonstrates the correlation between temperature changes throughout the seasons and the actual and potential yield of irrigated wheat. The graph illustrates that a substantial temperature contrast between summer and winter, i.e., seasonal fluctuations 3, results in a rise in both actual and potential yield. Cultivating wheat during autumn in such conditions elongates the growth period in contrast to warmer areas. Furthermore, a prolonged growth period owing to irrigation and water availability is a key aspect that contributes to the plant’s nutrient accumulation and ultimately enhances the yield. However, in rainfed cultivation where water resources are limited, a longer growth period may be a hindrance to the plant’s access to water, particularly during the seed filling stage [30]. Therefore, shortening the growth period to a range where rainfall is sufficient can help achieve potential yield [31]. Under these conditions, the highest potential and actual yield was obtained in seasonal temperature fluctuations 2. In rainfed conditions, access to water is crucial [32], and it is preferable to reduce the growth period (seasonal temperature fluctuations 1) rather than to lengthen it (seasonal temperature fluctuations 3).

According to the findings of this study, the potential for producing rainfed wheat globally is approximately 978 million tons, with a gap of 546 million tons. Similarly, the potential for irrigated wheat production worldwide is about 513 million tons, with a gap of 270 million tons. These results indicate a significant potential to increase wheat production globally, particularly in the neglected area of rainfed cultivation. In terms of irrigated cultivation, the North China Plain is a prime region for increasing production in both area and yield potential. Regarding rainfed conditions, the European continent is one of the primary areas globally with potential to increase wheat production.

## Conclusion

The top regions for irrigated wheat farming and production are located in the North China Plain, southern regions of the Tibetan Plateau, and along the banks of the Nile River. Suitable regions for irrigated wheat cultivation with high yield potential include the plains of North China, France, and central parts of the United States. Priority should be given to reducing the production gap in the main areas of irrigated wheat farming, such as China, the United States, Ukraine, and Iran. Rainfed wheat cultivation is mostly concentrated in various parts of the world, such as the northern areas of Kazakhstan in Asia, northern regions of Morocco in Africa, central parts of the United States in America, South-East and South-West of Australia in Oceania, and South-West of Russia and Ukraine in Europe. When it comes to rainfed wheat yield potential, the eastern parts of China are the most promising in Asia, while almost all regions in Europe show good potential. In Africa, Ethiopia is the most promising, while the northeastern United States and eastern Australia are the best in the American and Oceania continents, respectively. To address the production gap in rainfed wheat, the areas with the highest current cultivated area of this crop should be prioritized for gap reduction programs. Countries like France, Germany, England, and Poland with agricultural lands suitable for rainfed wheat production can also be prioritized for such programs.

The research had limitations, notably the absence of comprehensive global land use data for all crops and land uses. To overcome this challenge, we utilized the land use information of specific crops that are commonly rotated with wheat. Additionally, it is vital to emphasize the importance of the GYGA protocol as a valuable tool for evaluating yield potential and identifying yield gaps at various levels, including local, regional, and national scales. To improve our understanding of countries that have not been previously investigated, it is essential to collect more comprehensive data. This would facilitate more precise insights and informed decision-making on a global scale when conducting future investigations.

## Supporting information

S1 Fig Irrigated agricultural land (this area was obtained from the total area under cultivation of wheat, barley, chickpea, and rapeseed) (A), wheat cultivation area in the current condition (B), the area than can be considered to increase the area under wheat cultivation (the difference between A and B) (C) according to courtiers and climate zones. These maps are drawn in the main cultivation climates and in the courtiers that have the highest possibility of developing the cultivation area. Darker green color means increasing the surface area.
(DOCX)

S2 Fig Rainfed agricultural land (this area was obtained from the total area under cultivation of wheat, barley, chickpea, and rapeseed) (A), wheat cultivation area in the current condition (B), the area than can be considered to increase the area under wheat cultivation (the difference between A and B) (C) according to courtiers and climate zones. These maps are drawn in the main cultivation climates and in the courtiers that have the highest possibility of developing the cultivation area. Darker yellow color means increasing the surface area.
(DOCX)
