# Supplementary material for: Addressing food insecurity: An exploration of wheat production expansion
Source: PLoS One. 2023 Dec 13;18(12):e0290684. doi: 10.1371/journal.pone.0290684 (PMC10718460; doi:10.1371/journal.pone.0290684)
Supplement: S2 Fig — Rainfed agricultural land (this area was obtained from the total area under cultivation of wheat, barley, chickpea, and rapeseed) (A), wheat cultivation area in the current condition (B), the area than can be considered to increase the area under wheat cultivation (the difference between A and B) (C) according to courtiers and climate zones. These maps are drawn in the main cultivation climates and in the courtiers that have the highest possibility of developing the cultivation area. Darker yellow color means increasing the surface area. (DOCX) [file pone.0290684.s002.docx]

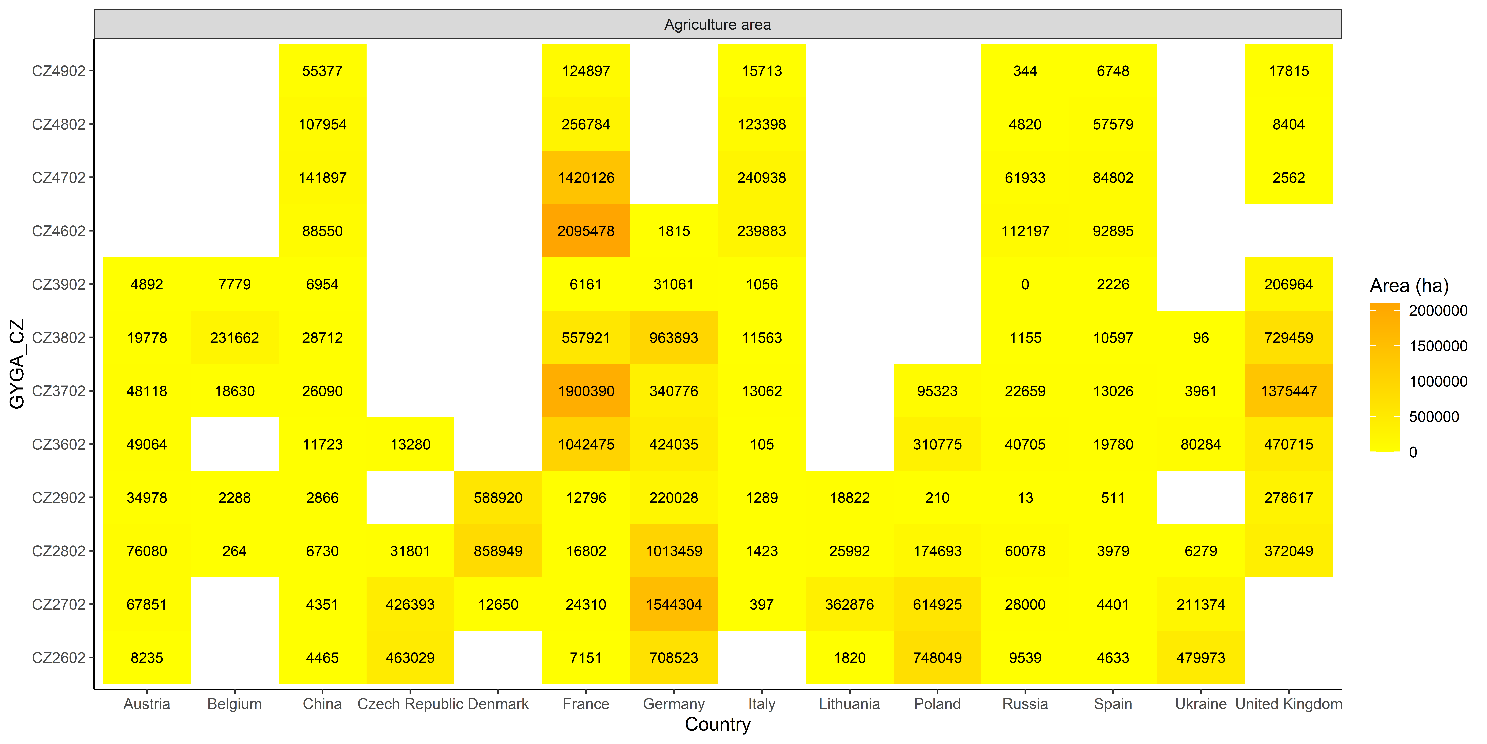


A

B


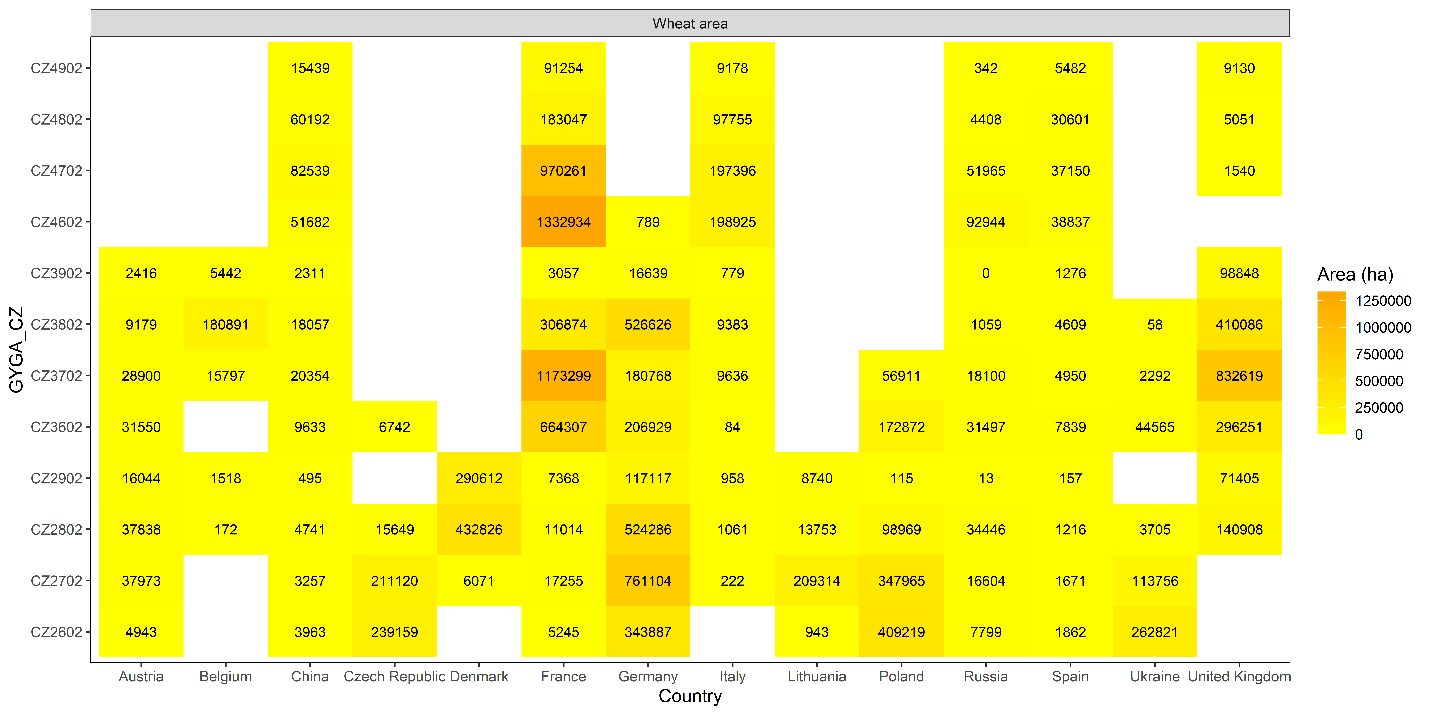


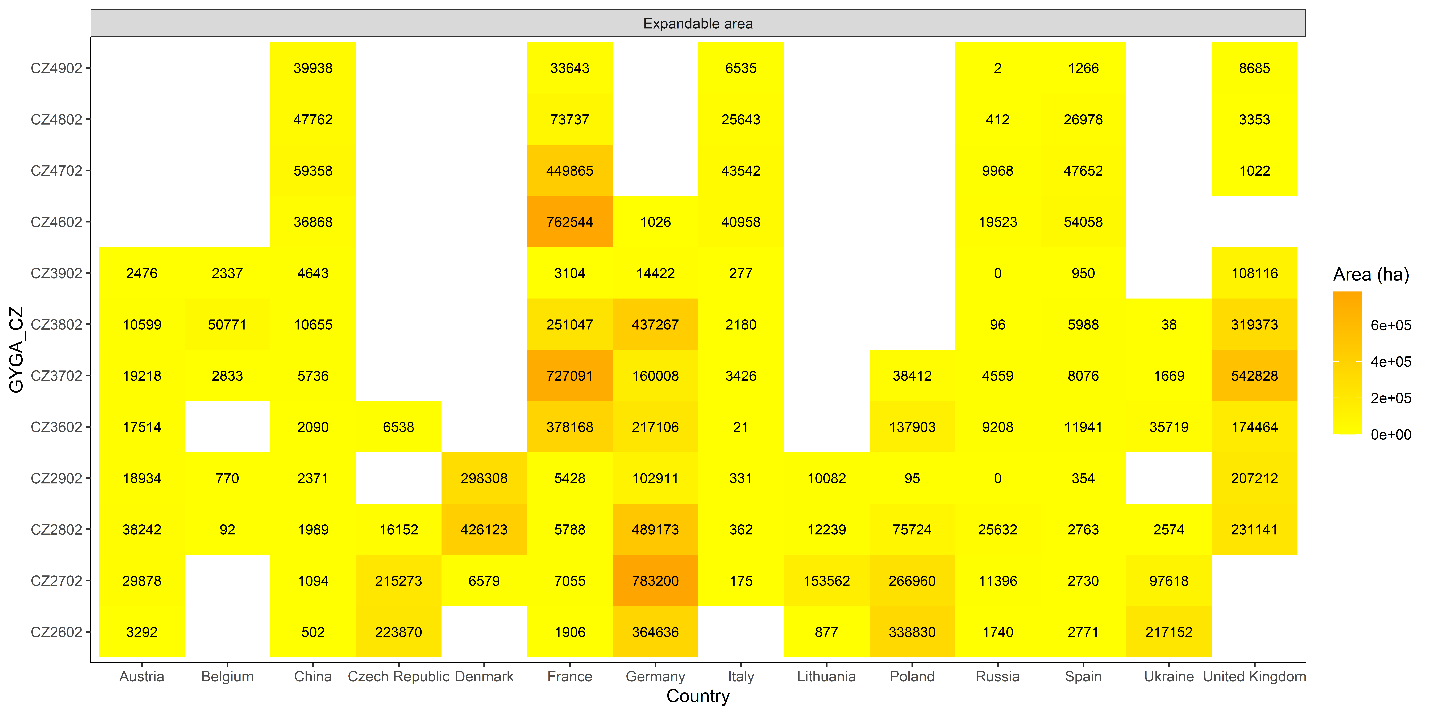
 S2 Fig. Rainfed agricultural land (this area was obtained from the total area under cultivation of wheat, barley, chickpea, and rapeseed) (A), wheat cultivation area in the current condition (B), the area than can be considered to increase the area under wheat cultivation (the difference between A and B) (C) according to courtiers and climate zones. These maps are drawn in the main cultivation climates and in the courtiers that have the highest possibility of developing the cultivation area. Darker yellow color means increasing the surface area.

C
